# Lymphatic drainage of sinonasal malignancies and the role of sentinel node biopsies

**DOI:** 10.1186/s13023-024-03127-8

**Published:** 2024-03-13

**Authors:** Fatemeh Kashani, BG Weiss, P Bartenstein, M Canis, F Haubner

**Affiliations:** 1 Department of Otorhinolarnygology, Head and Neck Surgery, Ludwig-Maximilians University, Marchioninistrasse 15, 81377 Munich, Germany; 2 Department of Nuclear Medicine, Ludwig-Maximilians University, Munich, Germany

**Keywords:** Sinonasal malignancy, Sentinel node biopsy, Head neck malignancies, Neck dissection, Lymph node scintigraphy

## Abstract

**Background:**

Locoregional recurrence is a critical factor in the prognosis of sinonasal malignancies. Due to the rarity of these tumours, as well as the heterogeneity of histologies and anatomical subsites, there is little evidence regarding the rate and location of regional metastases in sinonasal malignancies. Elective regional lymph node dissection in the therapy of sinonasal malignancies has become controversial. On the one hand, elective regional lymph node dissection is considered to be an overtreatment in the cN0 cases. On the other hand, undetected occult lymphatic metastases are associated with a poor prognosis. In this study, we discuss the role of sentinel lymph node biopsy as a minimally invasive procedure in the treatment of sinonasal malignancies based on our two years of practical experience and the currently available data.

**Results:**

This is a descriptive, monocentric, retrospective study, including 20 cases of cN0 malignant sinonasal neoplasm, that underwent a surgical therapy between 2020 and 2022. The following aspects were investigated: tumour entity, localisation of the primary tumour, tumoral stage, localisation of the sentinel lymph nodes, and postoperative complications. Squamous cell carcinoma was the most frequently diagnosed tumour entity (50%), followed by adenocarcinoma (20%) and malignant melanoma (15%), adenoid cystic carcinoma and mucoepidermoid carcinoma. Sentinel lymph nodes were most frequently found in the ipsilateral neck region I (45%), followed by the ipsilateral neck region II (40%). In all cases, the removed lymph nodes were free of malignancy. There were no postoperative complications due to lymph node biopsy. There were no recurrences during the study period.

**Conclusion:**

Sentinel node biopsy could add more safety to the management of cN0 sinonasal malignancies due to its low morbidity. Whether SNB could provide an alternative to elective neck dissection in the management of SNM should be investigated in further studies.

## Background

Sinonasal malignancies (SNM) are rare and heterogeneous tumours arising from the nasal cavity and paranasal sinuses. They account for approximately 3 to 5% of all head and neck malignancies and less than 1% of malignancies overall [1]. They cause few or no symptoms in the early stages of the tumour and tend to be locally advanced at the time of diagnosis. This leads to a poor prognosis (5-year overall survival for all SNMs 50–55%) [2], in spite of advanced treatments developed in recent decades. The clinical behaviour of the different histological types of SNM is highly variable, and each type is rare enough to make it difficult to develop an evidence-based approach to their management. Regional metastasis is a critical factor in the prognosis of sinonasal malignancies [3–5]. Clearly, patients with clinically positive lymph nodes require treatment of the neck. The treatment of patients with a cN0 neck is still controversial. There is currently no international guideline to recommend the management of these cases. On the one hand, undetected occult lymphatic metastases are associated with a worse prognosis [3, 6]. On the other hand, the invasive nature of elective neck dissection (END) increases the chance of surgery-related side-effects that impair the patient’s quality of life [7, 8]. END is therefore considered by many physicians to be an overtreatment with no survival benefit in the cases without lymph node metastases.

The complex lymphatic drainage pathways of the nasal cavity, paranasal sinuses, and neighbouring structures make the predicting of likely location of a metastasis a challenging aspect of the diagnosis and therapy of SNM. The sentinel lymph node (SLN) concept states that the spread of a malignant tumour is stepwise and embolic in nature, via the lymphatic vessels to the first-echelon lymph node(s). These lymph nodes are most likely to harbour occult metastases before tumour cells spread to further nodes. Excisional biopsy and pathological evaluation of the SLNs therefore allows prediction of the disease status of the remaining cervical lymph node basin, avoiding the need for a neck dissection and its morbidity and potential complications in the case of a negative result.

Sentinel node biopsy (SNB) is well studied in the management of breast carcinoma and malignant melanoma. SNB represents a highly accurate and considerably less-morbid staging method in breast carcinoma, which has replaced axillary lymph node dissection in early stages to avoid unnecessary axillary lymph node dissection and its morbidities [9, 10]. The advantages of SNB have also been shown in clinically localized melanoma, particularly in the cases of intermediate thickness [11].

In this study, we report our experience with sentinel node biopsy in 20 cases of sinonasal malignancy cases. We further discuss the potential benefits of sentinel node biopsy in the diagnosis and management of sinonasal malignancies.

## Results

Twenty patients (16 men and 4 women) were enrolled. The mean age was 65.2 years (range 53–84). Primary site, histological type of tumour, tumour stage and location of SLN are summarized in Table 1. Squamous cell carcinoma (SCC) was the most frequently diagnosed tumour entity (50%), followed by adenocarcinoma (20%) and malignant melanoma (15%). In all cases, a preoperative cN0 status was described. Sentinel lymph nodes were detected in the most cases in the ipsilateral neck region I (45%) and II (40%). In one case with a pT4 adenocarcinoma of the nasal cavity and lacrimal duct with orbital infiltration, the SLN was found in the ipsilateral preauricular region. In one other case with a pT2 mucoepidermoid carcinoma of the maxillary sinus, the SLN was found in the ipsilateral neck region III. In a patient with pT1 SCC of the nasal vestibule the SLNs were located bilaterally in the neck region I. In all cases, the removed lymph nodes were tumour-free. There were no postoperative complications (including haematoma, infection, abscess formation, wound healing disorders, shoulder dysfunction, facial nerve dysfunction and hypoglossal nerve dysfunction) due to lymph node biopsy. No regional recurrences were observed during the study period (median follow-up time 12.5 months).

**Table 1:** Disease and sentinel lymph node characteristics

Total patients	*N* = 20
Primary site	n (%)
- Nasal cavity	10 (50%)
- Nasal vestibule	7 (35%)
- Ethmoid sinus	2 (10%)
- Maxillary sinus	1 (5%)
Histological types	
- Squamous cell carcinoma	10 (50%)
- Adenocarcinoma	4 (20%)
- Malignant melanoma	3 (15%)
- Adenoid cystic carcinoma	1 (5%)
- Mucoepidermoid carcinoma	1 (5%)
- Undifferentiated carcinoma	1 (5%)
Pathological tumour category	
- T1	3 (15%)
- T2	11 (55%)
- T3	1 (5%)
- T4	5 (25%)
Clinical N category	
- cN0	20 (100%)
Pathological N category	
- pN0	20 (100%)
Location of sentinel lymph node	
ipsilateral	
- Neck region I	9 (45%)
- Neck region II	8 (40%)
- Neck region III	1 (5%)
- Preauricular	1 (5%)
bilateral	
- Neck region I	1 (5%)

## Discussion

For more than two decades, the sentinel node biopsy (SNB) technique has been the standard of care worldwide for the management of primary cutaneous melanoma, breast carcinoma, and cervical carcinoma. Long-term observations show an approximately fourfold higher complication rate after axillary dissection compared to SNB in the treatment of invasive breast carcinoma [9, 10], while the diagnostic accuracy of these procedures is comparable [12–15]. Recent studies have also demonstrated a comparable accuracy for SNB to selective neck dissection also in early oral and oropharyngeal squamous cell carcinoma [16–20]. In 2017, in a meta-analysis of 66 studies including more than 3500 patients with T1-2 cN0 oral squamous cell carcinoma (OSCC), SNB yielded a pooled sensitivity of 87% and a pooled negative predictive value of 94% [21]. den Toom et al [22] evaluated the diagnostic value of elective neck dissection (END) and SNB in early-stage OSCC in two large cohorts (390 (44%) END patients and 488 (56%) SNB patients) in 2020. They found an overall sensitivity of 84% in the END cohort and 81% in the SNB cohort, with a negative predictive value of 93% for both cohorts. In this study, SNB false-negative patients had almost the same disease-specific survival rate as true-positive patients. In contrast, the END false-negative cohort showed a dramatic decrease in survival compared to the END true-positive patients. Neck management in sinonasal malignancies has not been studied to the same extent. To our knowledge, there is no randomised controlled trial comparing the accuracy of SNB with END in the treatment of SNM. In the current study population, no initial lymph node metastases were diagnosed by SND and no late nodal metastases occurred during follow-up.

The main advantage of SNB over END is the less invasive nature of the procedure with a lower risk of postoperative morbidity and a better aesthetic result. The most common complications and morbidities after a neck dissection include hypo- or dysesthesia in parts of the neck or ear, neck pain and tension, shoulder discomfort, lymphedema, and cosmetic disfigurement [23]. In 2009 Schiefke et al [24] reported the results of a comparison of postoperative morbidity and health-related quality of life between patients underwent SNB and END for the treatment of the cN0 squamous cell carcinoma of the head and neck. Their conclusion was that functional outcome after sentinel node biopsy is significantly better than after elective neck dissection, although this difference was not reflected in the scores of the quality-of-life questionnaires. Functional status was assessed by scores for cervical scar, extent of lymphedema, sensory function, function of facial and hypoglossal nerve, cervical spine, and shoulder (Constant Shoulder Score [25]). Other studies have also shown a higher number of patients with neck haemorrhage requiring further drainage surgery, and a higher number of orocervical communications in the END compared to the SNB [26, 27]. This is consistent with the results of our study in SNM. We did not observed any postoperative complications due to lymph node biopsy.

A further advantage of SNB is shorter operative time and lower treatment costs. In 2013, Govers et al [28] published an analytical model for the management of the clinically N0 neck in T1 - T2 oral cancer to assess the cost-effectiveness of five strategies: END, wait and watch, gene expression profiling (GEP) followed by neck dissection or wait and watch, SNB followed by neck dissection or wait and watch, and GEP and SNB (for positive GEP) followed by neck dissection or wait and watch. In a similar study in 2016, van der Linden et al [29] analysed the cost utility of SNB compared to ultrasound-guided fine needle aspiration cytology (USgFNAC), USgFNAC and SNB (if USgFNAC negative) and END. Over a 5-year time horizon, SNB was the most cost-effective strategy per gained QALY (quality-adjusted life year) as compared to END in both trials. The therapy effects associated with SNB, and END were very similar in these trials (Table 2).

**Table 2:** Therapy effect a 5-year time horizon in the treatment of the cN0 neckin T1–T2 oral cancer

	SNB	END
Govers et al.	3.63 QALYs^*^	3.61 QALYs^*^
van der Linden et al.	3.70 QALYs^*^	3.67 QALYs^*^

* QALY, quality-adjusted life year

The heterogeneity in the clinical behaviour of the different histological types and anatomical subsites, as well as the rarity of SNM, limit an evidence-based consensus regarding the management of lymph nodes in SNM. However, the presence of regional metastases, including occult disease has been shown to have a significant impact on the prognosis of SNM [3–5]. Cantù et al [3] studied 704 patients with malignant tumours of the paranasal sinuses from 1968 to 2003. They reported a 2-year survival rate of 67.9% in patients with N0 ethmoid sinus tumours vs. 26.7% in those with N + tumours. The corresponding 5-year survival rates were 45.3% vs. 0%. For the maxillary sinus tumours, the 2-year survival rates were 70.3% vs. 48.5% and the 5-year survival rates were 50.6% vs. 16.8% [3]. The decision of when to treat regional lymph nodes is clear when there is evidence of nodal metastasis. Mirghani et al. summarized the recommendations of several authors for or against prophylactic neck treatment in a review [5]. Some authors see poor survival benefit for neck treatment in cN0 patients who have undergone appropriate staging investigations [30–32]. Others recommend a routine elective neck treatment for sinonasal squamous cell carcinoma and undifferentiated carcinoma depending on the T stage [33–37]. The aim of prophylactic neck treatment is to detect and eradicate microscopic metastases in patients without clinically and radiologically detectable lymph node metastases to reduce the risk of oncological neck failure. This approach is traditionally recommended when the risk of occult lymph node metastasis is 10–20% or greater [38]. In the literature, reported rates of neck relapse in SNM range from 2 to 33% [3–5, 32, 38, 39]. The correlation between primary site of tumour, histology, stage, or local extension and the occurrence of neck failures has been the subject of many studies. Carcinomas of the maxillary sinus, the most common site of SNM [36], have been shown to have a higher risk of regional metastasis [3, 6, 36]. In addition, several studies have reported that squamous cell carcinoma [3, 6, 33, 35, 36] and undifferentiated carcinoma [33, 35] are more likely to be associated with regional metastasis. In a recent study, Kayvan et al [40] found 14.1% occult nodal disease among 220 cN0 patients with the most common entity of SNM, squamous cell carcinoma. The relation between tumour stage and cervical metastasis is controversial. Some studies suggest that T1/T2 is more associated with neck metastases than T3/T4 [3, 6]. Conversely, in other studies, metastases were more commonly seen in advanced T stage due to local invasion into the orbit, dura, infratemporal fossa or palate [34]. These different results have been obtained over decades with varying accuracy of initial neck assessment [3, 6, 35, 36, 41]. In several studies, isolated neck recurrences could not be clearly distinguished from those associated with local failure [5]. The low incidence of SNM and the histological heterogeneity of these tumours also contribute to these inconsistent results.

In the clinically N0 neck, it is challenging not only to estimate the risk of occult nodal metastases but also to select which basins to treat. Few studies have investigated which lymph nodes in the neck are at highest risk in the setting of SNM. It has been shown that ipsilateral level II is the most common basin at risk, followed by level I of the ipsilateral neck [39, 42]. Fernández et al [39] found by lymphoscintigraphy during SNB in patients with sinonasal tumours that levels I to II most commonly contained the sentinel node [39]. This is consistent with the results of our study. Interestingly, we observed cases with the sentinel lymph nodes in the preauricular area and in the mandibular angle. These neck areas are usually not treated in prophylactic neck dissection, but may contain micrometastases. Due to the small patient population in this study, no significant conclusion can be drawn regarding the relationshiop between the characteristics of the primary and the location of the sentinel lymph node. However, this is an interesting aspect that requires further investigation.

It should be noted that there are several difficulties associated with lymph basin scintigraphy in head and neck malignancies. Due to the proximity of the injection site and the rapid flow of radiotracer through the lymphatic drainage pathway, the examination is time dependent [43, 44]. This can make it difficult to differentiate between the first and second echelon nodes. The size of the lymph node is also important. Small nodes can be very difficult to detect, especially in deeper areas of the neck, such as the parapharyngeal space. Furthermore, the anatomical accessibility of the tumour could certainly pose a challenge for the injection of the radiotracer. However, the tumour is usually easy to reach, as sinonasal tumours usually become symptomatic in later stages by nasal obstructing.

Due to the low prevalence of sinonasal malignancies and the great heterogeneity of these tumours, the number of cases in the studies is limited. This is also a limitation of this study. Our small study population doesn’t allow us to draw any significant conclusions about the accuracy and effectiveness of SNB in the treatment of cN0 SNM. Nevertheless, our results reflect the less invasive nature and reduced surgical trauma of SNB.

Regarding the advantages of SNB, there is a need for randomised controlled trials comparing the oncological outcomes of cN0 SNM patients undergoing SNB with other treatment strategies. The heterogeneity and rarity of SNM make this a challenging task.

## Conclusion

Sentinel node biopsy could add more safety to the management of cN0 sinonasal malignancies due to its low morbidity. Whether SNB could provide an alternative to elective neck dissection in the management of SNM should be investigated in further studies.

## Methods

This is a descriptive, monocentric, retrospective study. We reviewed 20 cases diagnosed as malignant neoplasm of the nasal cavity and paranasal sinuses (ICD-10 codes C30.0 and C31.-) that underwent surgical treatment between March 2020 and September 2022 at the Department of Otorhinolaryngology - Hospital of the Ludwig-Maximilian-University (LMU) Munich, an academic tertiary referral centre. The study was approved by the ethics committee of the Ludwig-Maximilians University (Munich, Germany) under the file number 21-1030. The ethics committee waived the need for informed consent since the study was limited to sole retrospective data collection; there were no changes in treatment caused by this study.

To detect the presence of metastatic lymph nodes, the initial systematic workup included a radiological assessment of the neck. All patients underwent contrast-enhanced computed tomography of the sinuses, neck and chest. Tumour stage was defined according to the 8th edition of the American Joint Committee on Cancer classifications [45]. All cases were discussed in a multidisciplinary tumour board. All patients were treated with curative intent.

**Fig. 1:**
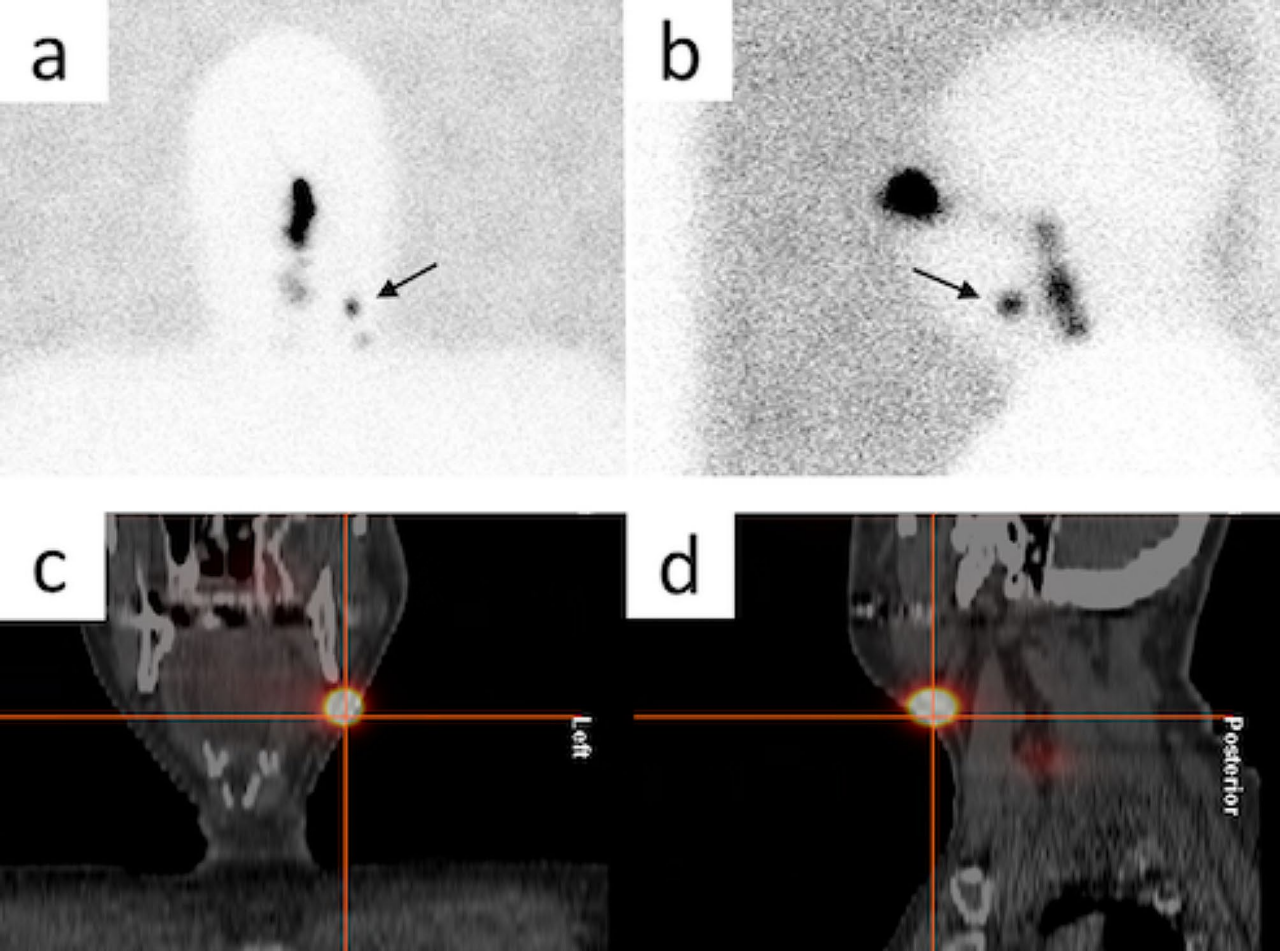
Sentinel node scintigraphy: (**a**) anterior and (**b**) lateral acquisition of static images, (**c**) and (**d**) SPECT/ldCT of the same SLN

For scintigraphy, a peritumoral injection of 0.05–0.2 ml saline solution of ^99m^Tc nanocolloidal with a total injected activity of 15–30 MBq was performed 24 h prior to surgery. The radiotracer was administered under endoscopy by two to four intramucosal injections, depending on the size and localization of the tumour, best in the cardinal points around the cancer, at 3, 6, 9, and 12 o’clock. The acquisition protocol included the following:

- Early static images: anterior and lateral acquisition within 5 min post-injection to visualize the lymphatic pathways, which makes it possible to distinguish between SLNs and second-tier lymph nodes. This step encompassed at least the first 10–15 min (Fig. 1a and b).
- Late static images: a late static image at 60–120 min post-injection using the same views as in the early static images to identify additional lymph nodes that receive a somewhat slower direct drainage from the tumour. Comparing early and late images provides a crucial tool that is needed to distinguish between the surgically relevant SLNs and irrelevant higher echelon nodes.
- Single-photon emission computed tomography/low-dose computed tomography (SPECT/ldCT): It should be performed immediately after the late static images. SPECT/ldCT provides accurate anatomical localization and depth evaluation of SLNs (Fig. 1c and d).

**Fig. 2:**
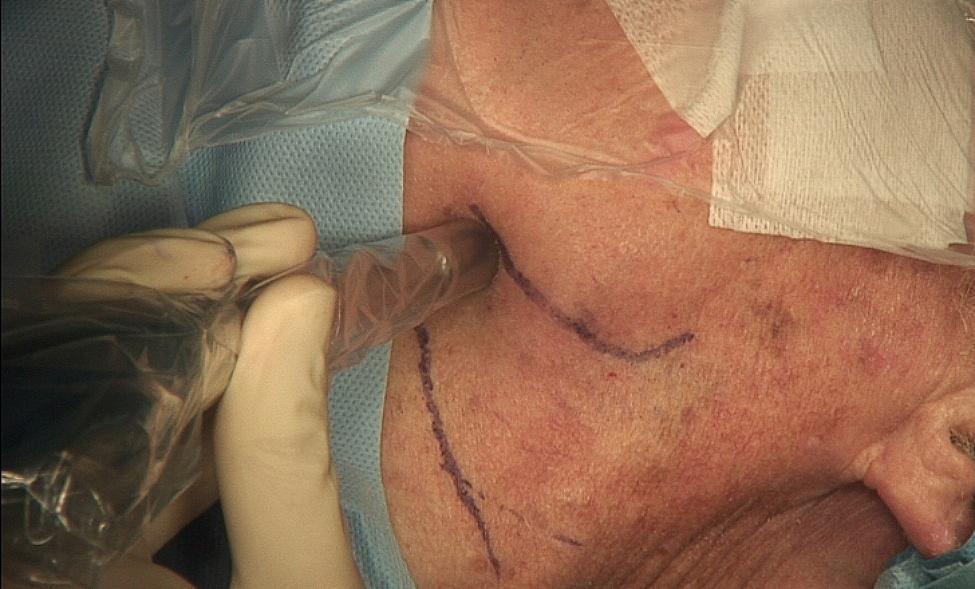
Detecting the SLN by a handheld gamma probe preoperatively

**Fig. 3:**
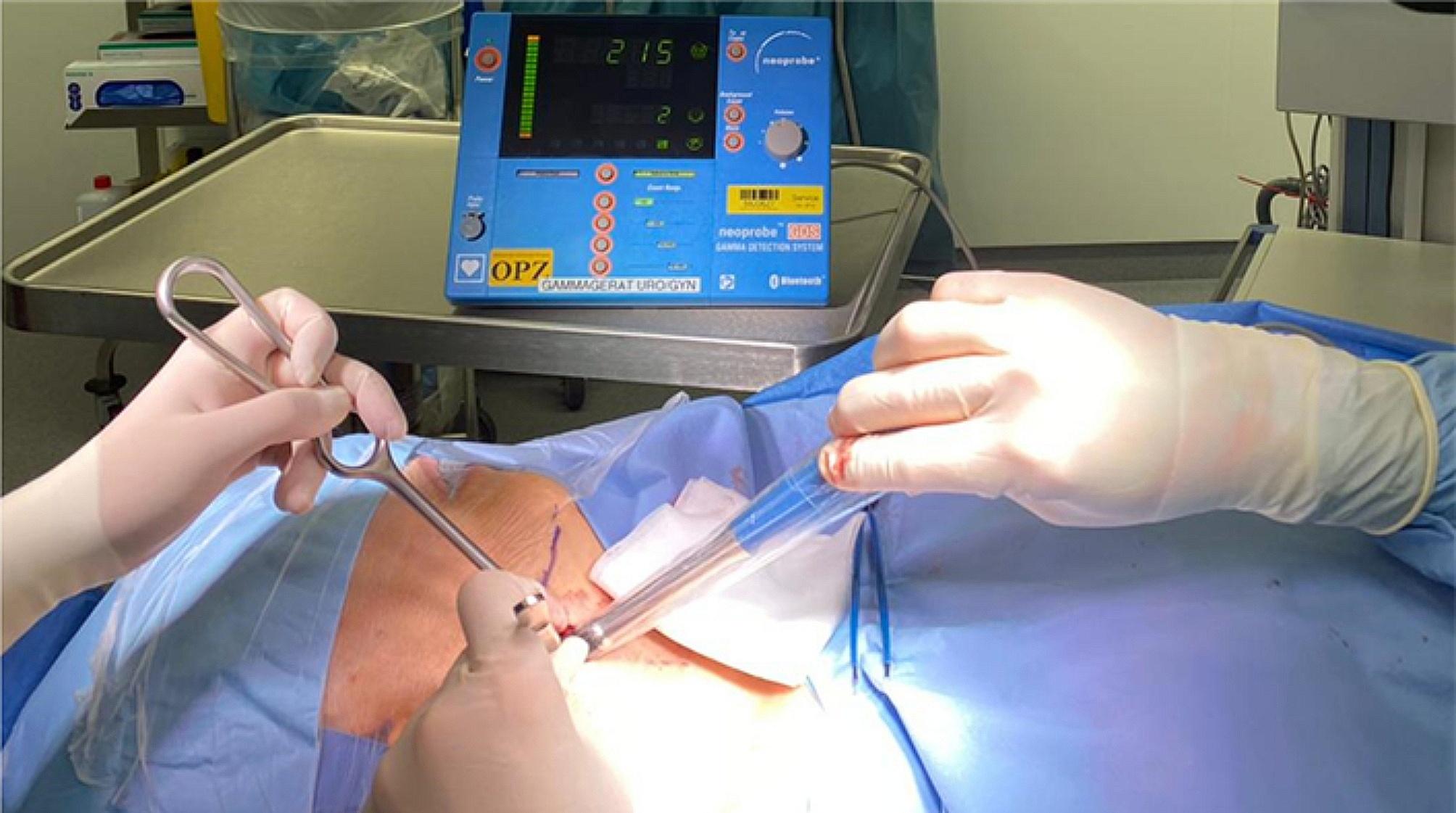
Detecting the SLN by a handheld gamma probe intraoperatively

A handheld gamma probe (Neoprobe® Gamma Detection System, Mammotome, Cincinnati, OH, USA) was used intraoperatively to detect the SLN (Figs. 2 and 3). The detected SLN was removed by means of a selective neck dissection of the corresponding neck region through a 2–3 cm Incision. The SLN-dissection in level I was performed under facial nerve monitoring. For confirmation, the removed lymph nodes were examined for positive extracorporeal gamma signal. An exact pathological examination followed.

Furthermore, the following aspects were investigated: Location of the tumour, tumour entity, tumour stage, location of the sentinel lymph nodes as well as possible postoperative complications such as hematoma, infection, abscess formation, wound healing disorder, shoulder dysfunction, facial nerve impairment and hypoglossal nerve dysfunction.

## Data Availability

The datasets used and analysed during the current study are not publicly available to preserve individuals’ privacy under the European General Data Protection Regulation. The datasets are available from the corresponding author on reasonable request.
